# Innate Immune Molecule Surfactant Protein D Attenuates Sepsis-induced Acute Pancreatic Injury through Modulating Apoptosis and NF-κB-mediated Inflammation

**DOI:** 10.1038/srep17798

**Published:** 2015-12-04

**Authors:** Zhiyong Liu, Qiao Shi, Jiao Liu, Osama Abdel-Razek, Yongan Xu, Robert N Cooney, Guirong Wang

**Affiliations:** 1 Department of Surgery, SUNY Upstate Medical University, Syracuse, NY, 13210, USA; 2 Department of Surgery, Wuhan University, Renmin Hospital, Wuhan 430060, Hubei Province, P.R.C

## Abstract

Sepsis causes multiple-organ dysfunction including pancreatic injury, thus resulting in high mortality. Innate immune molecule surfactant protein D (SP-D) plays a critical role in host defense and regulating inflammation of infectious diseases. In this study we investigated SP-D functions in the acute pancreatic injury (API) with C57BL/6 Wild-type (WT) and SP-D knockout (KO) mice in cecal ligation and puncture (CLP) model. Our results confirm SP-D expression in pancreatic islets and intercalated ducts and are the first to explore the role of pancreatic SP-D in sepsis. CLP decreased pancreatic SP-D levels and caused severe pancreatic injury with higher serum amylase 24 h after CLP. Apoptosis and neutrophil infiltration were increased in the pancreas of septic KO mice (p < 0.05, vs septic WT mice), with lower Bcl-2 and higher caspase-3 levels in septic KO mice (p < 0.05). Molecular analysis revealed increased NF-κB-p65 and phosphorylated IκB-α levels along with higher serum levels of TNF-α and IL-6 in septic KO mice compared to septic WT mice (p < 0.01). Furthermore, *in vitro* islet cultures stimulated with LPS produced higher TNF-α and IL-6 (p < 0.05) from KO mice compared to WT mice. Collectively, these results demonstrate SP-D plays protective roles by inhibiting apoptosis and modulating NF-κB-mediated inflammation in CLP-induced API.

Sepsis, the host response to infection, is considered to be the major cause of death in severe infections. More than 120,000 patients die of sepsis each year in the United States alone^1^. Despite of our advancements in medical technology, well-equipped intensive care units and better practice treatments^2^, the rate of sepsis-related mortality remains in excess of ~30%^3^,^4^. Severe sepsis can lead to multiple organ dysfunction^5^. The development of organ dysfunction is highly correlated with increased mortality. The more organs that fail, the higher the mortality^6^. Although the most common dysfunctions in septic patients are the lung and kidney^1^, the pancreas is also vulnerable to inflammation and injury in the septic patients^7^ and animal models of sepsis induced by CLP^8^.

Surfactant protein D (SP-D), a member of C-type lectin family, plays an important role in host defense and regulating inflammation during infections^9^. Although SP-D is predominantly expressed in the lung, it is also found in extrapulmonary tissues/organs such as kidney^10^, human nasal epithelium^11^, the digestive tract and mesentery^12^, the lacrimal system^13^, human salivary glands and saliva^14^. The functions of SP-D in extrapulmonary tissues are poorly understood^15^. SP-D is composed of four functional domains, including N-terminal cysteine-rich domain, collagen-like domain, neck region and carbohydrate recognition domain (CRD)^16^. The CRD of SP-D can bind to the carbohydrate molecules on the surface of various microbes (such as viruses, bacteria, yeast and fungi), and enhance macrophages and neutrophils to take in the attachment of microorganisms, as well as facilitate the clearance and killing^17^. In normal situation, SP-D adheres to signal regulatory protein-α (SIRP-α) of inflammatory cells by CRD to prevent the release of inflammatory cytokines^17^. However, when pathogenic microbes invade, CRD binds to microbial carbohydrates, the collagenous tail interacts with CD91 of inflammatory cells to stimulate NF-κB activation, which induces the production of inflammatory cytokines^18^,^19^. For these reasons, we hypothesized that SP-D has a role in the pathogenesis of sepsis-induced acute pancreatic injury (API).

SP-D-mediated immune responses have been studied using various pathogenic organisms in SP-D-null mice. Collectively, these provide evidence that SP-D has a protective function in various infections, because the SP-D knockout (KO) mice show higher susceptibility to bacterial and viral pathogens^20^,^21^. SP-D KO mice also demonstrate increased inflammation and pulmonary injury caused by LPS^22^. Recent data from our laboratory provide evidence that SP-D plays protective roles on indirect kidney injury induced by CLP-induced sepsis^23^.

The current study examines the hypothesis that SP-D is expressed in the pancreas and plays a protective role in sepsis-induced API. This study identifies and verifies SP-D expression in the pancreatic islets and intercalated ducts of mice using SP-D KO mice as a negative control. Using the cecal ligation and puncture (CLP) sepsis model to cause API in SP-D KO and wild type (WT) mice we found that SP-D play a protective role in the sepsis-induced API by modulating NF-κB-mediated inflammation and inhibiting apoptosis.

## Results

### Expression and localization of SP-D in the islets and the intercalated ducts of mouse pancreas

**Figure 1:**
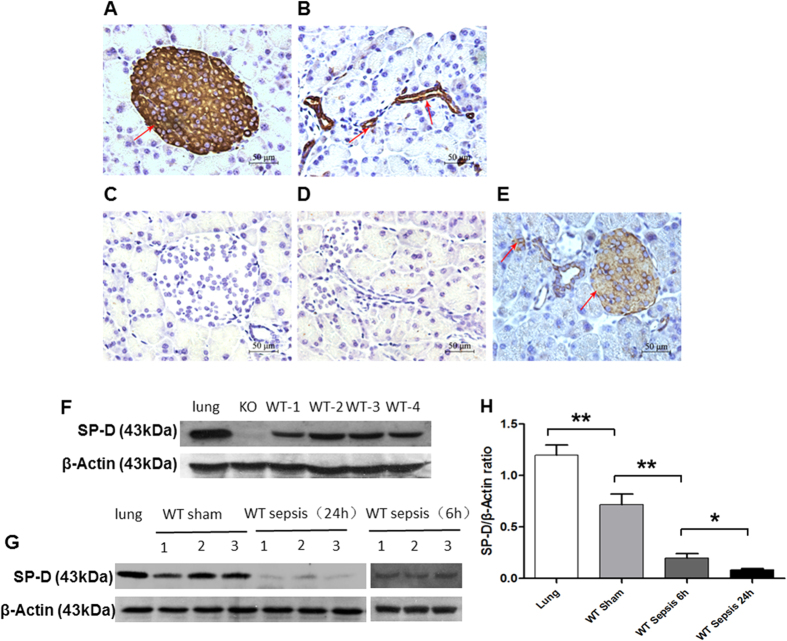
Analysis of surfactant protein D (SP-D) expression in the pancreas. SP-D expression was analyzed by IHC (**A–E**) and Western blotting (**F,G**) methods. Pancreatic islets (**A**) and the intercalated duct system (**B**) stained positive for SP-D expression. As expected, there was no SP-D expression in the islet (**C**) and the intercalated duct system (**D**) of the pancreas in KO mice. Decreased SP-D expression was detected in the pancreatic islets and the intercalated duct system of septic mice (**E**). The results from Western blotting analysis show SP-D expression was observed in the pancreas of WT mice, but not SP-D KO mice (**F**). Furthermore, the levels of SP-D in the pancreas of septic WT mice 6 and 24 hrs after CLP and Sham controls were examined by Western blotting analysis. The results demonstrate decreased SP-D level in the pancreatic tissues of septic WT mice compared to Sham WT mice (p < 0.01) (**G,H**). Lung tissue was used as a positive control, (Magnification x400, scale bars = 50 μm).

We examined expression and localization of SP-D in the mouse pancreas by Immunohistochemical and Western blotting analyses. As shown in Fig. 1A,B, SP-D was expressed and localized in the islets and the intercalated ducts of pancreas by Immunohistochemistry (IHC). A negative control with SP-D-null mice is shown in Fig. 1C,D, and decreased SP-D level was observed in the pancreas of septic mice (Fig. 1E). To confirm the expression of SP-D protein, we performed Western blotting analysis with total proteins from the WT mouse pancreas. As shown in Fig. 1F, the expression of SP-D was found in the pancreas of WT mouse, using lung as positive control and SP-D-null mouse pancreases as negative control. Furthermore, in CLP-treated mice, the levels of SP-D expression were significantly decreased 6 and 24 hrs after CLP compared with the untreated animals (Fig. 1G,H).

### Acute pancreatic injury in septic SP-D KO and WT mice

**Figure 2:**
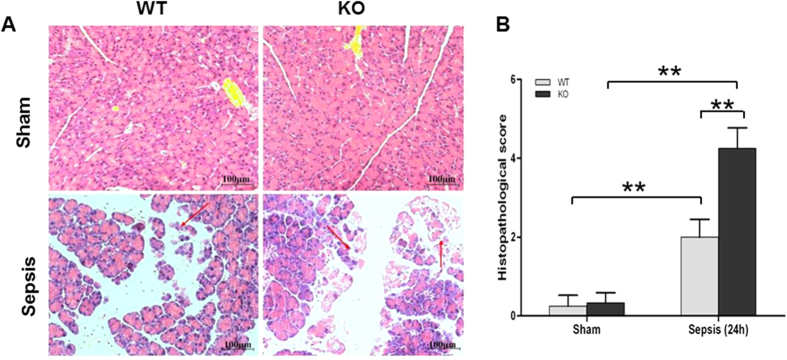
Histopathology of the pancreas in septic WT and KO mice. Pancreatic tissues from WT mice and SP-D KO mice were examined under the light microscope after staining with H/E (**A**). The histopathological scores of pancreatic injury were assessed (**B**). The pancreatic tissues in septic WT and SP-D KO mice showed severe pathological injury 24 h after CLP. Inflammation, edema, and necrosis of the acinar cells were observed. Pancreatic histopathological score index in septic SP-D KO mice was higher than that of septic WT mice (**B**). magnification × 200. Graphs represent the mean ± SEM. **p < 0.01. scale bars = 100 μm. (n = 6 mice/group).

Previous studies have shown the pancreas is vulnerable to injury during sepsis^24^. Therefore, we examined histopathological changes in pancreatic tissues from septic SP-D KO, WT and sham mice. As shown in Fig. 2, there were obvious pathological changes in the septic SP-D KO and WT mice 24 h after CLP treatment, but not in control mice (Fig. 2A). The pancreatic tissues in both septic KO and WT mice exhibited characteristic edema, inflammation and necrosis of the acinar cells, but more severe injury was found in the SP-D KO mice compared with WT mice (Fig. 2A). Quantitative analysis of the pancreatic histopathological score revealed that septic SP-D KO mice was higher compared with septic WT mice 24 h after CLP (Fig. 2B, p < 0.01), suggesting that SP-D KO mice were more sensitive to sepsis-induced API.

### Increased serum amylase activity in septic SP-D KO and WT mice

**Figure 3:**
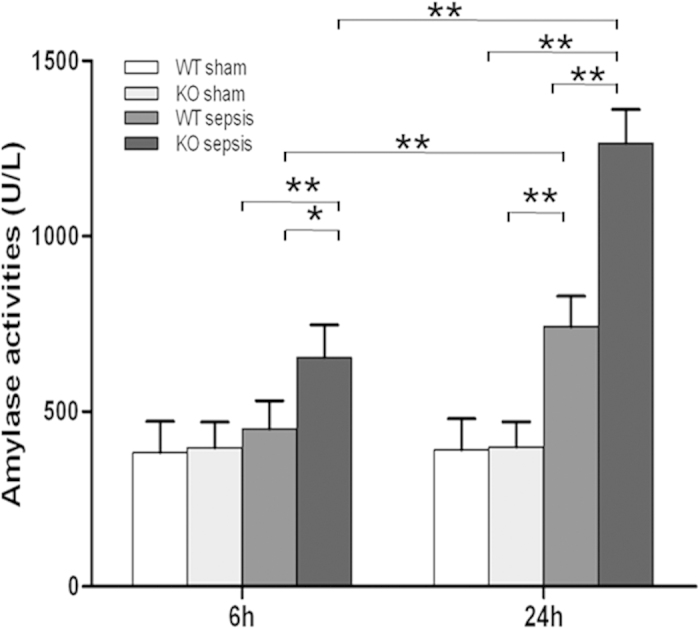
Amylase activity in the serum of septic WT and SP-D KO mice. Serum samples were collected from WT and SP-D KO mice 6 h and 24 h after CLP or sham operation. The amylase activity was determined by amylase activity assay kit. The amylase level at 6 h and 24 h after CLP were significantly elevated in SP-D KO mice compared with WT mice. Graphs represent the mean ± SEM. *p < 0.05, **p < 0.01. (n = 6 mice/group).

Serum amylase activity is a commonly used biomarker to detect pancreatic injury. Amylase activity was slightly increased at 6 h post-CLP in the WT than sham mice, but it was higher in the septic SP-D KO mice compared to sham and WT mice (p < 0.05) (Fig. 3). Amylase activity was dramatically increased at 24 h for septic SP-D KO and WT mice compared to sham mice (p < 0.01). Septic SP-D KO mice demonstrate a higher level of amylase activity than septic WT mice (p < 0.01) (Fig. 3).

### Apoptosis of the pancreas in septic SP-D KO and WT mice

**Figure 4:**
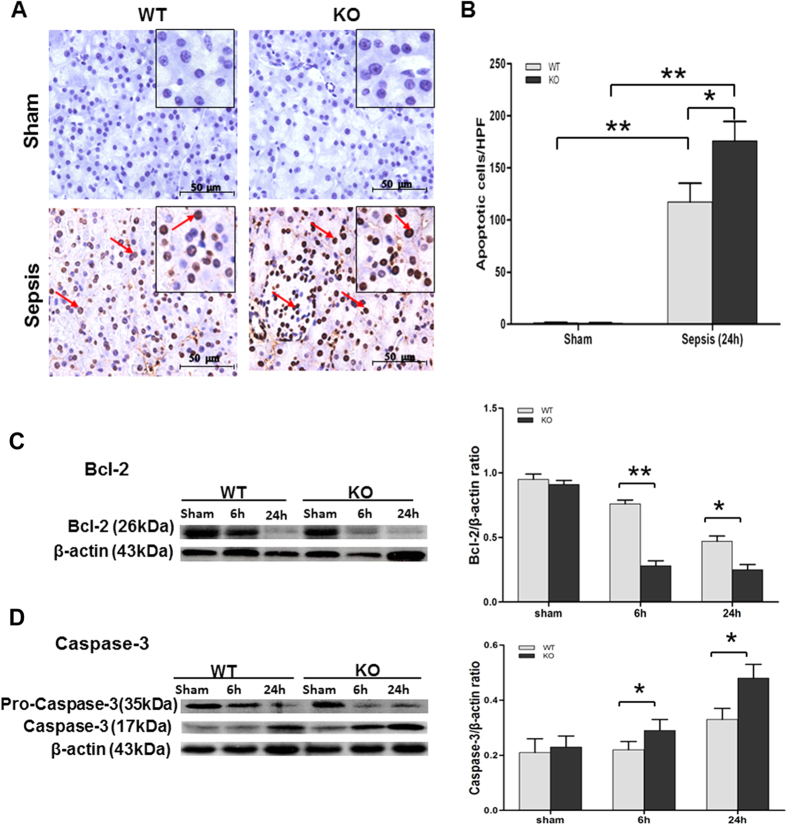
Apoptosis in the pancreas of septic WT and SP-D KO mice. Apoptotic cells were examined by the TUNEL analysis (**A,B**) in the pancreas of septic WT and SP-D KO mice 24 h after CLP or sham (**A**). Apoptotic cells with brown nucleus were observed (arrows). Apoptotic cells were quantified by 8 randomly high power fields (HPF) at x400 magnification. The number of apoptotic cells was larger in septic SP-D KO mice than septic WT mice 24 h after CLP (**B**). Furthermore, the levels of two apoptotic biomarkers (Bcl-2 and caspase-3) were analyzed in the pancreas of septic WT and SP-D KO mice by Western blotting method. β-actin was used as an internal control to normalize protein levels. Lower Bcl-2 level (**C**) and higher activated caspase-3 (17 kDa band) level (**D**) were observed in the pancreas of septic SP-D KO mice compared with septic WT mice 6 h and 24 h post-CLP. Graphs represent the mean ± SEM, *p < 0.05, **p < 0.01. Scale bars = 50 μm. (n = 6 mice/group).

To explore the role of SP-D in pancreatic cell apoptosis in sepsis-induced API, the slides of pancreas were examined by the TUNEL method. As shown in Fig. 4, a number of apoptotic cells (including the pancreatic acinar cells, intercalated ducts cells, as well as some types of cells in islets) with brown nucleus were observed in the pancreases of septic, but not in sham mice. Quantitative analysis of apoptotic cells demonstrates that the number of TUNEL-positive cells was higher in pancreas from septic SP-D KO mice compared with septic WT mice 24 h after CLP (Fig. 4B, p < 0.01).

To investigate potential mechanisms for SP-D effects in sepsis-induced apoptosis, two apoptosis-related biomarkers were measured by Western blotting analysis in the pancreatic tissues of septic and sham mice. As shown in Fig. 4C,D, the expression levels of Bcl-2 or caspase-3 showed differences between septic SP-D KO mice and septic WT mice. Bcl-2 is an inhibitor of apoptosis and was used as a negative biomarker. The expression level of Bcl-2 was decreased in septic compared with sham mice (p < 0.01), but the amount of Bcl-2 in septic SP-D KO mice was lower than septic WT mice 24 h post-CLP (Fig. 4C, p < 0.01). In contrast, the expression level of caspase-3 (17 kDa band as active form), a biomarker of apoptosis, was increased in septic mice compared with sham mice; and the level of caspase-3 was higher in septic SP-D KO mice than septic WT mice at 6 h (p < 0.05) and 24 h (p < 0.05) after CLP (Fig. 4D). These results provide evidence SP-D plays a role in inhibiting apoptosis during sepsis-induced API.

### Neutrophil infiltration in the pancreas of septic SP-D KO and WT mice

**Figure 5:**
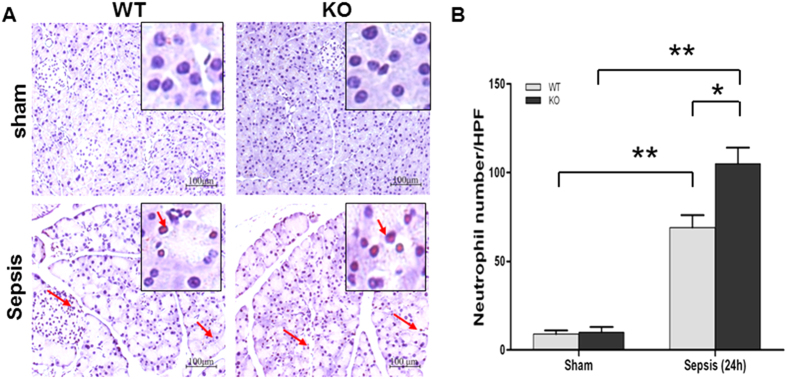
Neutrophil infiltration in the pancreatic tissue of septic WT and KO mice. A large number of neutrophils infiltrated into pancreatic tissues in septic WT and KO mice (**A**) (Arrow). Quantification of neutrophils was determined by 8 randomly high power fields (HPF) at x200 magnification. Compared with septic WT mice, the number of neutrophils was larger in septic SP-D KO mice 24 h post-CLP (**B**). Graphs represent the mean ± SEM, *p < 0.05, **p < 0.01. Scale bars = 100 μm. (n = 6 mice/group).

Inflammatory cell infiltration is also an important mechanism in the pathogenesis of sepsis-induced API. To evaluate pancreatic neutrophil infiltration, we used neutrophil-specific antibody to perform IHC analysis of pancreatic tissue. The results show that pancreas from septic SP-D KO mice display more neutrophil positive cells than septic WT mice (Fig. 5A). Quantitative analysis of neutrophils by light microscopy indicates the number of neutrophils in pancreas from septic SP-D KO mice increased significantly compared with septic WT mice (Fig. 5B). These data indicate SP-D inhibits neutrophil infiltration into septic pancreases.

### NF-κB activation in the pancreas of septic SP-D KO and WT mice

**Figure 6:**
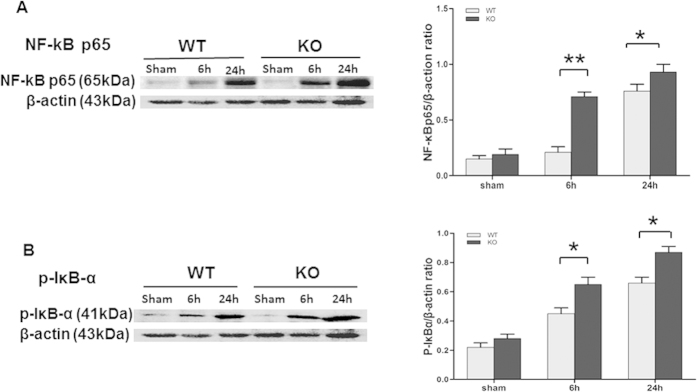
Levels of NF-κB p65 and phosphorylated IκB-α (p-IκB-α) in the pancreas of septic WT and SP-D KO mice. The levels of NF-κBp65 and p-IκB-α were analyzed by Western blotting method. β-actin was used as an internal control to normalize protein levels. NF-κB p65 (**A**) and p-IκB-α (**B**) levels were significantly increased in the pancreas of septic SP-D KO mice compared to septic WT mice 6 h and 24 h post-CLP. Graphs represent the mean ± SEM, *p < 0.05, **p < 0.01. (n = 6 mice/group).

Previous studies by Yoshida *et al.*^18^ demonstrated SP-D is involved in the regulation of NF-κB signaling pathway. Phosphorylation of IκB-α is required for initiation of NF-κB activation. Therefore, the levels of phosphorylated IκB-α (p-IκB-α) and NF-κB p65 in the pancreas were examined by Western blotting analysis. As shown in Fig. 6, there were significant increases of the levels of p-IκB-α and NF-κB p65 in septic mice 12 and 24 h after CLP compared with control animals (Fig. 6, p < 0.01). Importantly, the levels of p-IκB-α and NF-κB p65 in the pancreas from septic SP-D KO mice were higher when compared with the pancreas from septic WT mice (Fig. 6, p < 0.05). These results indicated treatment with CLP in SP-D-null mice resulted in enhanced activity of NF-κB as compared with septic WT mice.

### Pro-inflammatory cytokine levels in the sera of septic SP-D KO and WT mice

**Figure 7:**
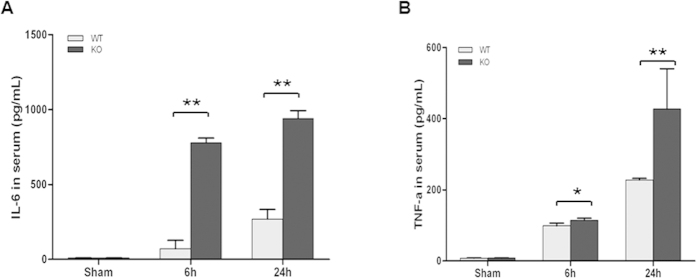
Pro-inflammatory cytokine levels in the sera of septic WT and SP-D KO mice. Pro-inflammatory cytokines IL-6 and TNF-α in the sera from septic and sham mice were determined by ELISA. The levels of IL-6 (**A**) and TNF-α (**B**) in the sera of septic WT and SP-D KO mice significantly increased at 6 h and 24 h as compared with controls (sham mice). The levels of IL-6 and TNF-α were higher in septic SP-D KO mice than septic WT mice 6 h and 24 h post-CLP. Graphs represent the mean ± SEM. *p < 0.05, **p < 0.01. (n = 6 mice/group).

To evaluate systemic inflammation, the levels of circulating pro-inflammatory cytokines IL-6 and TNF-α in the serum were measured by ELISA. As shown in Fig. 7A,B, CLP resulted in significant expression of IL-6 and TNF-α in the sera of septic mice at 6 h and 24 h after surgery compared with sham mice. Furthermore, the levels of IL-6 and TNF-α were higher in the sera of septic SP-D KO mice than in septic WT mice (Fig. 7A,B, p < 0.05).

### Productions of pro-inflammatory cytokines in the cultured islets stimulated with LPS

**Figure 8:**
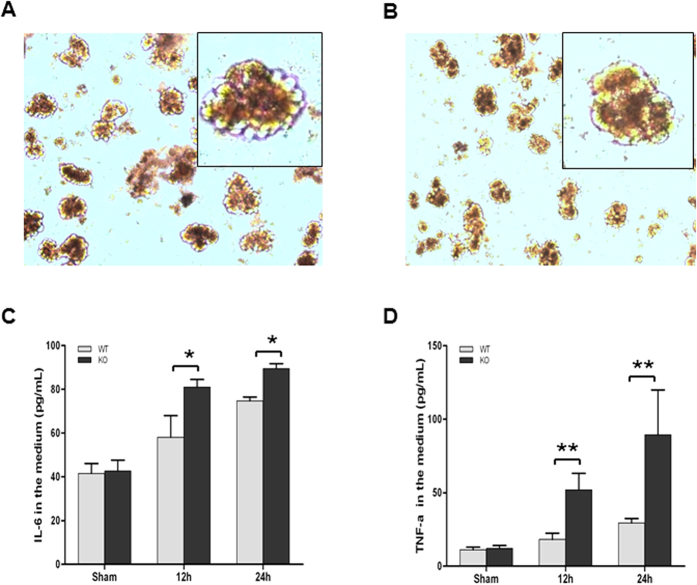
Pro-inflammatory cytokine production in the isolated islets in stimulation with LPS. Panels (**A**,**B**) show the isolated islets before and after LPS stimulation, respectively. The concentrations of pro-inflammatory cytokines (IL-6 and TNF-α) in the conditioned media from islet cultures with stimulation of LPS were determined by ELISA. The levels of IL-6 (**C**) and TNF-α (**D**) in the septic WT and SP-D KO mice significantly increased at 12 h and 24 h after stimulation with LPS as compared with controls. The levels of IL-6 and TNF-α were significantly increased at 12 h and 24 h after LPS stimulation in the conditioned medium of islets of SP-D KO mice compared to WT mice. Graphs represent the mean ± SEM. *p < 0.05, **p < 0.01. (n = 3 independent experiments).

In order to investigate the regulation of inflammation in pancreas by SP-D we isolated and cultured pancreatic islets from SP-D KO and WT mice. Cultured islets were stimulated by adding LPS (5 μg/mL) into the media (Fig. 8A,B). The phenotypes of cultured islets showed some differences before (Fig. 8A) and after (Fig. 8B) LPS stimulation. Twenty four hrs after LPS stimulation most islets from both SP-D KO and WT mice became smaller cell aggregates and exhibited apoptotic cell fragments in the culture medium compared to untreated islets. Pro-inflammatory cytokines IL-6 and TNF-α were measured in the conditioned media from islet cultures. After 12 h and 24 h exposure to LPS (5 μg/mL), the levels of IL-6 and TNF-α were markedly increased (Fig. 8C,D) than sham group, and the levels of IL-6 and TNF-α were higher in the conditioned medium of islets from SP-D KO mice compared to WT mice (Fig. 8C,D). These data suggested that SP-D can directly play a protective role in the local islet tissue to regulate pro-inflammatory cytokine production.

## Discussion

Previous studies have demonstrated that SP-D is expressed in the epithelial cells of mucosa surfaces in humans^10^. SP-D expression was also detected in several other organs and tissues from fetuses, children, and adults^25^. We are among the groups to confirm SP-D expression in pancreas and to localize SP-D expression in the islets and the intercalated ducts of mouse pancreas^25^,^26^. Our IHC analysis clearly demonstrates SP-D expression in the islets with SP-D KO mouse control. While the exact role(s) of pancreatic SP-D are poorly defined, in pregnancy, increased pancreatic SP-D expression correlates with increased ß-cell proliferation^26^. SP-D is known to play a crucial role in lung surfactant homeostasis^27^, innate host defense and modulating inflammation during pulmonary infections^9^,^28^. However, the results of the recent study indicate SP-D has a protective role during sepsis-induced API. These findings are consistent with the anti-inflammatory effects of SP-D in described endothelial cells^29^. They are also consistent with anti-inflammatory role posited for SP-D in response to cytokine stimulation of pancreatic islets^26^. Furthermore, cellular and molecular analyses revealed the mechanisms of SP-D through inhibiting pancreatic cell apoptosis and regulating the NF-κB-mediated inflammation in sepsis-induced API.

In the present study, we found that CLP-induced sepsis caused remarkable pancreatic injury 24 h after treatment in the septic mice compared with sham mice. Characteristic edema, inflammation, and necrosis of the acinar cells were observed in the pancreas by histopathological analysis, and pancreatic injury was worse in septic SP-D KO mice compared with septic WT mice. Although variation of histological changes in the pancreas of patients reported^30^, the results of this study clearly indicate increased susceptibility of SP-D KO mice to sepsis-induced API.

Serum amylase level is a biomarker which is commonly used to detect acute pancreatic injury^31^,^32^. Pancreatic blood supply can be affected earlier in sepsis than other organs^33^ thus increasing the possibility of pancreatic injury. In our study, serum amylase level is increased at 6 h after CLP, and significant increase in amylase activity in the serum occurred at 24 h in both WT mice and SP-D KO mice, with higher activity in septic SP-D KO vs. septic WT mice. These demonstrated that CLP-induced sepsis resulted in severe pancreatic injury 24 h after CLP; and SP-D expression in the pancreas and other organs may protect from sepsis-induced API. In the present study, we observed decreased SP-D level in the pancreas in septic mice 6 and 24 h post-CLP. The observations might be explained due to apoptosis and necrosis of a larger number of SP-D expressing cells in the pancreas of septic mice. Decreased SP-D level in the pancreas further lost SP-D protective roles in the host defense and anti-inflammatory regulation, as well as apoptosis^34^. Indeed, SP-D protective effects were also observed in other organs, like kidney^23^ and liver^35^, in the early stage of sepsis-induced dysfunction.

Apoptosis has been found in clinical and experimental API^36^. In the present study, a number of apoptotic cells were observed in the pancreas of septic mice at 24 h after CLP. In previous studies, mild acute pancreatitis was found to be associated with extensive apoptosis while severe acute pancreatitis was noted to involve extensive severe necrosis but with a little apoptosis^37^,^38^. It was also found that induction of apoptosis around the necrotic tissues can reduce the severity in severe AP^39^. This study has observed only moderate necrosis in sepsis-induced acute pancreatic injury of both SP-D KO and WT mice but many apoptotic cells and increased apoptosis-related biomarkers were found. Moreover, septic SP-D KO mice showed more severe pancreatic injury compared to septic WT mice. Therefore, these indicate excessive apoptosis may be harmful to the function of the pancreas. Apoptosis is a form of programmed cell death, it is the consequence of several interlinked intracellular caspase proteins^40^,^41^. Caspase proteins belong to a family of intracellular cysteine protease, they play important roles in the initiation and execution of apoptosis. Both Bcl-2 and caspase-3 are important regulatory proteins of apoptosis. Bcl-2 acts as an inhibitor of apoptosis, overexpression of Bcl-2 will inhibit apoptosis. Caspase-3 is an executor of apoptosis, overexpression of caspase-3 will accelerate apoptosis^42^,^43^. In this study, apoptotic cells were identified in the pancreas evidenced by the changed levels of apoptosis regulatory proteins in the pancreas. These indicate that apoptosis of pancreatic cells occurred at the early phase of API in this study, which is consistent with other studies^36^,^44^. In addition, we observed that septic SP-D KO mice showed more severe apoptosis compared with septic WT mice, suggesting that SP-D had a protective effect to pancreatic apoptosis in sepsis-induced API. These findings were consistent with protective effects of SP-D against renal apoptosis in sepsis-induced acute kidney injury (AKI)^23^.

Nuclear factor-kappa B (NF-κB) is a transcription factor that regulates the expression of many inflammatory genes in various infectious diseases^45^. In the pancreas, NF-κB is a p65/p50 heterodimer as a predominant form^46^. Under normal conditions, NF-κB dimers are blocked nuclear localization by inhibitory proteins (IκBs) and are trapped in the cytoplasm as inactivated forms. When cells are stimulated with various factors, the inhibitory proteins are phosphorylated, which allows the NF-κB to translocate into the nucleus, where it binds to relevant consensus sequence of NF-κB target genes, rendering NF-κB activation^47^. Activated NF-κB can induce many genes transcription, including inflammatory and apoptotic responses^48^. In this study, both NF-κB p65 and p-IκB-α levels in the pancreas were significantly increased in the septic compared to sham mice 6 h and 24 h post-CLP, but septic SP-D KO mice have more NF-κB and p- IκB-α expression than septic WT mice. These data suggest that SP-D could modulate NF-κB activation during the early phase of sepsis-induced API. Spontaneous activation of NF-κB has been observed in alveolar macrophages of SP-D-null mice^18^ and SP-D acts as an important modulator of pulmonary inflammation^49^. Recent studies suggested increased caspase-3 expression is positively associated with the NF-κB p65 level in kidney tissues from septic patients^50^. Previous studies have shown that SP-D can bind to TLR4^51^ and inhibit TLR4-mediated pro-inflammatory responses^52^. The loss of functional TLR4 lead to a blunted NF-κB response, reduced pro-inflammatory mediator production, and decreased sensitivity to hyperoxia in mice^53^. Collectively, the findings from this and previous studies provide solid evidences that SP-D can ameliorate sepsis-induced API through regulating NF-κB-mediated signaling pathway and inhibiting pancreatic apoptosis.

Inflammatory cell infiltration may also play a key role in the pathogenesis of sepsis-induced API. Neutrophil infiltration contribute to parenchymal cell injury and vascular dysfunction during liver injury^54^. Our IHC studies reveal intensive neutrophils in the interstitial capillaries and the pancreatic tissue in septic WT and SP-D KO mice. In contrast to septic WT, neutrophil infiltration increased significantly in the septic SP-D KO mice. Previous work found that SP-D KO mice were more susceptible to LPS-induced acute lung injury compared to WT mice, and intratracheal instilled SP-D can inhibit lung inflammation^55^.

Pro-inflammatory cytokines are important mediators in the pathogenesis of API^56^. As expected, in the present study CLP sepsis induced a serious systemic inflammatory response with significant increase of both pro-inflammatory cytokines TNF-α and IL-6 in the serum 6 h and 24 h after CLP. These data are supported by the increased NF-κB and p-IκB-α levels in the pancreas of septic WT and SP-D KO mice. Our findings are consistent with previous studies that demonstrated increased TNF-α and IL-6 in the serum during polymicrobial sepsis by CLP^57^,^58^. Additionally, *in vitro* experiments with isolated islet cultures stimulated by LPS demonstrated SP-D function in the modulation of inflammatory cytokine production in the islet cells.

In summary, our results demonstrate SP-D expression in pancreatic islets and intercalated duct. SP-D can decrease pro-inflammatory cytokine production and amylase activity in the serum, and inhibits pancreatic apoptosis thus attenuating API in the mouse sepsis model. The cellular and molecular mechanisms include SP-D inhibition of apoptosis and modulation of NF-κB-mediated inflammation in the sepsis-induced API.

## Materials and Methods

### Animals

All of the experiments in this study were approved by Institutional Animal Care and Use Committee at SUNY Upstate Medical University (IACUC #270) and meet the National Institutes of Health and ARRIVE guidelines on the use of laboratory animals. The original SP-D KO mice (C57BL/6 background) were kindly provided by Dr. Hawgood’s laboratory of the University of California, San Francisco. The SP-D KO mice were backcrossed at least 10 generations with C57BL/6 background mice^59^,^60^. All SP-D KO mice used in this study were bred in the animal core facility at SUNY Upstate Medical University^61^. Age-matched C57BL/6 WT mice were purchased from Jackson Laboratories (Bar Harbor, ME). There were no significant difference in phenotype between SP-D KO and matched WT mice. Age, sex and background-matched mice were housed in a temperature-controlled room at 24 °C under the pathogen-free conditions, maintained on water and food *ad libitum* before the study. Both male and female mice are used in this study.

### Pancreatic islet isolation and culture

Islets from C57BL/6 WT and SP-D KO mice were isolated by collagenase digestion as described previously^62^. In brief, the pancreas of mouse was distended by injecting 3 ml collagenase VI solution (0.5 mg/ml)(Sigma, St. Louis. MO), and then digested in a water bath at 37^o^ C for 15 min. The islets were washed with Hank’s solution, the collected islets were cultured in RPMI-1640 (Gibco, Grand island, NY) containing 10% FBS, 100 U/ml streptomycin and penicillin. Isolated islet cultures were exposed for 12 h and 24 h in the presence or the absence of LPS (5 μg/mL).

### CLP model

Cecal ligation and puncture (CLP) was performed to induce polymicrobial sepsis as previously described^23^,^63^. In brief, mice (8–12 weeks) were anesthetized with intraperitoneal ketamine/xylazine (90 mg/kg ketamine, 10 mg/kg xylazine) injection. After the indicative of anesthsia, under aseptic conditions, a ventral midline incision (1-cm) was performed, the cecum was exposed, then ligated with a 5–0 silk suture at 1.3 cm position from distal to the base of the cecum, but avoiding intestinal obstruction. The cecum was punctured twice using a 22-gauge needle at top and bottom, respectively. The punctured cecum was squeezed to extrude about 1-mm^3^ droplet of fecal material from the perforation sites and returned to the peritoneal cavity. The incision was closed in layers with 5–0 surgical sutures. Immediately after the procedure, mice were injected with prewarmed sterile saline (1 ml) subcutaneously to replace fluid lost. Pain medication was injected every 12 hrs after surgery (buprenorphine 0.05 mg/kg). For sham animals, mice underwent the same procedure, but no CLP.

### Tissue harvesting

Mice were anesthetized, and sacrificed at 6 h and 24 h after CLP or sham surgery as previously described^23^,^63^. Blood samples were collected from the inferior vena cava by means of 1-ml syringe, and plasma was collected after centrifugation (4000 xg) at 4^o^ C for 5 min. Pancreatic tissues were removed and immediately frozen in liquid nitrogen or fixed in 10% formalin for further study. Cultured Islets treated with or without LPS (5 μg/ml) were collected by centrifugation (15000 x g, 5 min) at 4^o^ C, and immediately frozen at −20^o^ C.

### Histopathological analysis

Formalin-fixed pancreatic tissues were embedded in paraffin and sectioned at about 4-μm for routine histology from six mice for each group^23^. Slides were stained with hematoxylin and eosin (H&E). Histopathology of the pancreatic injury was qualitatively evaluated by two experienced investigators who were blinded as to the experimental groups. Pancreatic histological grading was scored according to the method described by Demols *et al.*^64^.

### Biochemical measurement

Serum amylase (AMY) activity was measured to monitor the pancreatic injury by using a commercial kit (Sigma-Aldrich, St. Louis. MO). Levels of AMY were determined according to the manufacturer’s instructions.

### Apoptotic cell determination

A TUNEL kit (Roche, Indianapolis, IN) was used to detect apoptosis in pancreatic cells from septic and sham mice as the described previously^23^. In brief, sections from paraffin-embedded pancreas were incubated at 60 °C for 20 min, then deparaffinized and rehydrated. Sections were digested for 10 minutes with 2 μg/mL proteinase K, washed with PBS, incubated in 25 mmol/L cobalt chloride and so on according to the manufacturer’s protocol. Apoptotic cells were quantified at high-power field (x 400 magnification) by Nikon ECLIPSE TE2000-U optical microscopy.

### Immunohistochemistry (IHC)

The parrffin-embedded pancreas specimens were sectioned about 4μm for (IHC). Briefly, sections were de-waxed and rehydrated and put in 3% H_2_O_2_ to block endogenous peroxidase activity for 10 minutes. Epitope retrieval was carried out by boiling in 10 mM citrate buffer (pH = 6.0) for 20 min. After the sections were incubated with 10% goat serum for 40 minutes at room temperature, they were added with SP-D antibody (1:500, Santa Cruz Biotechnology, Dallas, Texas) or Anti-Mouse neutrophils antibody (1:100, Hycult Biotech, the Netherlands) at 4 °C overnight. Subsequently, the sections were applied biotinylated secondary antibody (Vector Laboratories, Burlingame, CA) at 1:2000 dilution and incubated in a humidified chamber at 37 °C for 30 minutes. DAB was used as a chromogenic substrate to incubate for 3–10 minutes, and then the sections were counterstained for 1–3 minutes with hematoxylin. Slides were viewed by an Olympus microscope (Olympus AG, Zurich, Switzerland). The positive cells were counted at high-power field (x 200 magnification).

### Western blotting analysis

Frozen pancreatic tissues were homogenized in RIPA cell lysis buffer containing PMSF (Thermo Scientific Pierce, Rockford, Illinois) and cocktail of protease inhibitors (Roche, Indianapolis, IN), as well as aprotinin (Sigma-Aldrich, St. Louis, MO). All samples were centrifuged at 14,000xg for 10 min. Supernatant was recovered, and protein concentration of the lysate was determined by using a bicinchoninic acid (BCA) protein assay kit (Thermo Scientific, Rockford, IL)^65^. Total protein (100 mg) for each sample were resolved by reducing, electrophoresed on 12% SDS-PAGE gel electrophoresis and transferred onto PVDF membranes (Bio-Rad, Hercules, USA). The membrane was soaked in Tris-buffered saline (TBS) containing 5% defatted milk for 1 hour, and was then incubated with a primary antibody against SP-D (Santa Cruz Biotechnology, Dallas, Texas), or caspase-3 (Santa Cruz Biotechnology, Dallas, Texas), or Bcl-2 (Santa Cruz Biotechnology, Dallas, Texas), or p-IκB-α (Santa Cruz Biotechnology, Dallas, Texas), or NF-κB p65 (Santa Cruz Biotechnology, Dallas, Texas) at 4 °C overnight. A β-actin antibody (Santa Cruz Biotechnology, Dallas, Texas) was used as internal control. After washing the membranes, a HRP-conjugated secondary antibody (Bio-Rad, Hercules, CA) was applied and incubated at room temperature for 1 hour. Washed again, the blots were exposed to X-ray film (Pierce Biochemicals, FL) for 2–3 min. The relative expression of protein was quantified after scanning of the films by the Quantity One software (Bio-Rad, Hercules, CA).

### Cytokine determination in the serum and the conditioned media from islet cultures

The concentrations of IL-6 and TNF-α in serum and conditioned medium of islet cell cultures were measured using commercially available murine enzyme-linked immunosorbent assay (ELISA) kits in accordance with the manufacturer’s instructions (Life Technologies, Frederick, MD)^23^.

### Statistical analysis

All the data are expressed as mean ± SEM. Results were analyzed by one-way analysis of variable (ANOVA) and Tukey’s post-hoc tests with the Sigmastat software version 3.5 (Jandel Scientific, CA). If P values were less than 0.05, differences were considered as statistically significant.

## Additional Information

**How to cite this article**: Liu, Z. *et al.* Innate Immune Molecule Surfactant Protein D Attenuates Sepsis-induced Acute Pancreatic Injury through Modulating Apoptosis and NF-κB-mediated Inflammation. *Sci. Rep.* **5**, 17798; doi: 10.1038/srep17798 (2015).
